# Intermittent fasting alleviates ulcerative colitis via lithocholic acid-mediated macrophage reprogramming

**DOI:** 10.3389/fnut.2026.1841890

**Published:** 2026-05-25

**Authors:** Yujen Tseng, Lingxi Lin, Feng Ji, Huan Song, Lin Chen, Maolin Ye, Suhan Zhao, Qi Zhou, Shaocong Mo, Xiangxi Ye, Jie Liu, Wanwei Zheng, Feifei Luo

**Affiliations:** Department of Digestive Diseases, National Clinical Research Center for Aging and Medicine, Huashan Hospital, Fudan University, Shanghai, China

**Keywords:** intermittent fasting, lithocholic acid, macrophage, metabolic reprogramming, ulcerative colitis

## Abstract

**Background:**

Ulcerative colitis (UC) is a relapsing inflammatory disorder, in which nutritional intervention has emerged as a modifiable factor for influencing disease onset and severity. This study aimed to investigate whether intermittent fasting (IF) alleviates UC and identify the underlying mechanisms, focusing on bile acid metabolism-immune system crosstalk.

**Methods:**

The study employed a 5:2 IF intervention (5 days *ad libitum* eating, 2 days low-calorie intake) in a chronic colitis model. Bile acid metabolic flux and lithocholic acid (LCA) levels were measured via metabolomics profiling, while gut microbiota composition was analyzed via 16S rRNA sequencing. Mechanistic studies explored the effect of LCA on macrophage polarization and metabolic reprogramming. UC patient samples were integrated to validate correlations between LCA levels, disease severity, erythrocyte sedimentation rate (ESR) and calprotectin.

**Results:**

5:2 IF promoted remission of chronic colitis. Mechanistically, IF reshaped gut microbiota composition, enhanced bile acid metabolic flux and elevated LCA levels. LCA directly inhibited pro-inflammatory macrophage polarization via inducing metabolic reprogramming and enhancing mitochondrial oxidative respiration. Analysis of human UC samples revealed that higher LCA levels correlated with milder UC, lower ESR, and reduced calprotectin.

**Conclusions:**

IF alleviated chronic experimental colitis by enhancing bile acid metabolism and elevating LCA, which exerted anti-inflammatory effects via regulating macrophage mitochondrial oxidative respiration. This identifies LCA as a key mediating metabolite and provides a mechanistic basis for understanding how IF may affect intestinal inflammation in preclinical models.

## 1 Introduction

Ulcerative colitis (UC) is a subtype of inflammatory bowel disease (IBD) characterized by chronic inflammation and ulceration of the innermost lining of the colon and rectum. The clinical course of UC is unpredictable, marked by alternating periods of exacerbation and remission (1). Despite recent advancements in biological therapies, the primary response rate to these treatments remains modest, typically ranging from 50%−60%, with only 15%−20% of patients achieving sustained remission (2–5). Furthermore, long-term maintenance of remission remains a significant challenge, as disease flare-ups occur in 55% of patients over a 10-year follow-up period (6). As a result, there has been increasing interest in nutritional interventions as both treatments and adjunct therapies for UC (7, 8).

Intermittent fasting (IF) dictates a dietary strategy of alternating caloric restriction and feeding window, which stimulates adaptive cellular responses. Accumulating evidence has continued to demonstrate the benefits of IF on a broad spectrum of disease including obesity, cardiovascular diseases, cancers, neurological disorders and autoimmune diseases (9–11). These include enhanced glucose regulation, improved stress resistance, and reduced inflammation (12). However, the beneficial effects remain empirical, while the underlying molecular mechanism remains unclear, especially in UC.

Bile acids, cholesterol-derived metabolites traditionally recognized for their roles in lipid digestion and energy homeostasis, have emerged as pivotal regulators in intestinal immunity and inflammatory diseases. Synthesized in the liver as primary bile acids, these molecules undergo extensive microbial modifications in the gut, including deconjugation, dehydroxylation, and isomerization, to generate secondary bile acids such as lithocholic acid (LCA), deoxycholic acid (DCA), and ursodeoxycholic acid (UDCA) (13, 14). Additionally, bile acid metabolism is highly responsive to dietary composition and undergoes significant alterations in response to changes in diet.

The present study explores the effect of 5:2 IF regimen in a dextran sulfate sodium salt (DSS) induced chronic colitis animal model (15). Results demonstrated that IF accelerated colitis recovery in murine models. Mechanistically, IF induced remission via an LCA-associated pathway by remodeling gut microbial composition, which enhanced oxidative phosphorylation in macrophages, thereby suppressing pro-inflammatory macrophage polarization. This multilevel regulation provides a mechanistic explanation for the beneficial effects of IF in UC.

## 2 Materials and methods

### 2.1 Animals

Six-week-old C57BL/6 mice were purchased from Southern Model Biotechnology, Shanghai. All mice were housed in the specific pathogen-free (SPF) level animal facility. The environment in the animal facility was maintained at a constant temperature and humidity (23°C, 50% humidity) and followed a 12-h light and 12-h dark cycle. Both male and female mice were used. Mice were randomly assigned to experimental groups using a computer-generated random number sequence. All *in vivo* assessments (body weight, DAI score, colon length, histological scoring) were performed by an investigator blinded to the group allocation. The study protocols involving mice were reviewed and approved by the Fudan University Institutional Animal Care and Use Committee (IACUC Approval No. 202501002S).

### 2.2 IF intervention

The mice received 5:2 IF fasting regimen, which involved 5 days of *ad libitum* feeding followed by 2 days of fasting. The mice were fed on a standard chow diet, which had a total energy content of 3,800 kcal/kg with 21.3% kcal protein, 11.4% kcal fat, and 67.3% kcal carbohydrates. On fasting days, the mice received 1 g of food per day, equivalent to 30% of their normal daily intake. To ensure accurate monitoring of food intake and to prevent competition or aggressive behavior, each mouse was housed individually in separate cages during fasting intervention.

### 2.3 DSS-induced chronic colitis in mice

To induce chronic colitis model in mice, 2.5% DSS was administered in the drinking water. Mice were given the DSS solution for 6 or 7 days, followed by a recovery period with regular drinking water for 7 days. This cycle was repeated for a total of two cycles to establish chronic colitis. Male and female mice were both used to establish the disease.

### 2.4 Antibiotic treatment

Mice were given a cocktail of antibiotics in their drinking water. The cocktail consisted of 0.5 g/L ampicillin, 0.5 g/L neomycin, 0.25 g/L vancomycin, and 0.25 g/L metronidazole. Mice received antibiotic treatment 14 days before DSS administration.

### 2.5 LCA intervention

Mice received 2.5% DSS in drinking water for 6 or 7 days, followed by a 7-day recovery period with regular drinking water. This cycle was repeated once. During each 7-day recovery period, mice were administered 40 mg/kg of LCA (Sigma) once daily via oral gavage. LCA was dissolved in a vehicle solution composed of 10% DMSO, 30% PEG8000, 10% Tween80, and 50% water. The control group received gavage of vehicle.

### 2.6 DAI and modified DAI

Mice were monitored daily for body weight changes, stool consistency and rectal bleeding. DAI score combined all three aspects, while modified DAI only contains stool consistency and rectal bleeding, considering fasting intervention contributes to significant weight changes. The scoring system is as follow: for weight loss, 0 for < 1%, 1 for 1%−5% loss, 2 for 5%−10% loss, 3 for 10%−20% loss, and 4 for more than 20% loss. For stool consistency, 0 for normal pellets, 1 for soft pellets, 2 for loose stool, 3 for mild diarrhea, 4 for severe diarrhea. For rectal bleeding, 0 for no bleeding, 1 for minimal color change, 2 for positive hemoccult test, 3 for visible blood in stool, and 4 for gross bleeding from anus with clotting.

### 2.7 H&E staining

To assess the severity of colonic inflammation, Swiss rolls were prepared from colon tissues and stained with hematoxylin and eosin. Fresh colon tissues were rinsed with cold phosphate buffered saline (PBS). Swiss rolls were prepared by fixing the tissue in 10% formalin, embedding in paraffin, and sectioning for staining.

Histological scoring was performed based on three parameters: ulcer count, epithelial changes, and inflammatory infiltration. For ulcer count: 0 for normal, 1 for one ulcer, 2 for two ulcers, 3 for three ulcers, and 4 for more than three ulcers. For epithelial changes: 0 for normal epithelium, 1 for focal goblet cell loss, 2 for extensive goblet cell loss, 3 for crypt loss, and 4 for extensive crypt loss. For inflammatory cells infiltration: 0 for no infiltration, 1 for infiltration confined to the crypt region, 2 for infiltration extending into the muscularis mucosae, 3 for widespread infiltration in the muscularis mucosae, and 4 for infiltration reaching the submucosa.

### 2.8 Culture and polarization of BMDMs

Bone marrow cells were flushed out of the bones using a syringe filled with sterile PBS and passed through a 70 μm cell strainer obtain a single cell suspension. Isolated cells were cultured with RPMI-1640 with 10% fetal bovine serum (FBS), 1% penicillin-streptomycin, 1% GlutaMAX and 20 ng/ml macrophage-colony stimulating factor (M-CSF) to promote differentiation. Replace the medium every 2 days with fresh medium containing M-CSF. Six days later, adherent cells were stimulated with 100 ng/ml LPS or 20 ng/ml IL-4 to induce the polarization.

### 2.9 RNA-seq

Total RNA was extracted from BMDMs using RNAmini kit (Qiagen). The quality and concentration of RNA were evaluated and measured using the Qubit Fluorometer (Thermo Fisher Scientific). Strand-specific libraries were created using the TruSeq RNA Sample Preparation Kit (Illumina) and sequenced on an Illumina NovaSeq 6000 system. Sequencing produced 2 × 150 bp paired-end reads. Differential expression analysis was conducted using the DEGseq package. *P*-values were adjusted for multiple comparisons using the Benjamini–Hochberg method.

### 2.10 Quantitative real-time PCR

Total RNA was extracted from cultured BMDMs using the NcmSpin Cell/Tissue Total RNA Kit (NCM biotech) according to the manufacturer's instructions. RNA concentration and purity were assessed using a NanoDrop 2000 spectrophotometer (Thermo Fisher Scientific). Reverse transcription was performed using the PrimeScript™ RT Master Mix (Takara Bio). Real-time PCR was carried out using ChamQ Universal SYBR qPCR Master Mix (Vazyme) on a QuantStudio Real-Time PCR System (Thermo Fisher Scientific). The primer sequences used are listed in Table 1.

**Table 1:** Primer sequence.

Primer	Sequence (5^′^–3^′^)
*Il10* Forward	CTTACTGACTGGCATGAGGATCA
*Il10* Reverse	GCAGCTCTAGGAGCATGTGG
*Tgfb1* Forward	CCACCTGCAAGACCATCGAC
*Tgfb1* Reverse	CTGGCGAGCCTTAGTTTGGAC

### 2.11 Metabolomics mass spectrometry analysis

Metabolomic profiling was carried out using ultra-high-performance liquid chromatography (UHPLC) coupled with a high-resolution mass spectrometer (HRMS). Distal colonic tissues were collected from mice for subsequent metabolomic analysis. An Acquity UHPLC system (Waters Corporation) was employed for chromatographic separation. Mass spectrometry analysis was conducted on a Q Exactive Orbitrap mass spectrometer. Data processing and metabolite identification were carried out using Compound Discoverer software (Thermo Fisher Scientific). Metabolite features were identified by matching retention times and mass spectra to a reference library. The relative quantification of metabolites was performed by comparing the peak areas of detected compounds across samples. Metabolite levels between the two groups were compared using the Mann-Whitney U test. *P* values were adjusted for multiple comparisons by the Benjamini-Hochberg false discovery rate (FDR) method.

### 2.12 Flow cytometry

Centrifuge the cell suspension and resuspend the cell pellet to perform Fc receptor blocking by incubating the cells with anti-CD16/32 antibody. After blocking, incubate the cells with fluorochrome-conjugated antibodies against surface markers for 30 min at 4°C. Wash the cells twice with PBS to remove unbound antibody. Permeabilize the fixed cells using IC Fixation for 20 min at room temperature followed by intracellular staining. The percentage of CD86 and iNOS positive cells was calculated based on FMO-cutoffs in the macrophage gate (F4/80^+^ CD11b^+^), where the positive gate was set to less than 1% of events in the FMO control. At least 30,000 events per sample were acquired on Beckman CytoFLEX and analyzed using FlowJo v10.

### 2.13 Microbiome analysis

Microbial samples were collected from the feces of mice. Total genomic DNA was extracted using DNA extraction kit (Qiagen). Polymerase chain reaction was performed to amplify the target regions. Unique dual-index barcodes were added to each sample using a second round of PCR to enable multiplex sequencing. The final library was quantified using qPCR or fluorometry to ensure optimal concentration for sequencing. Equimolar amounts of each sample library were pooled to create a single sequencing library. The pooled library was loaded onto an Illumina MiSeq platform for paired-end sequencing. Differential abundance of taxa between groups was assessed using DESeq2. The Benjamini–Hochberg procedure was used to control the false discovery rate (FDR). To predict the functional profiling of the gut microbiota and investigate the mechanistic link to lithocholic acid (LCA) production, functional inference analysis was performed using PICRUSt2 based on the 16S rRNA gene sequencing data. Subsequently, the R package ggpicrust2 was utilized to convert the KO abundance profiles into KEGG pathway abundances.

### 2.14 Seahorse

Seahorse XF Analyzer (Agilent) was employed to assess cellular bioenergetics in macrophages. Briefly, cells were seeded in XF 96-well microplates and incubated overnight at 37°C with 5% CO_2_. The XF Cell Mito Stress Test Kit was utilized to sequentially inject oligomycin (1 μM), FCCP (1 μM), and rotenone/antimycin A (0.5 μM) to measure key parameters of oxidative phosphorylation.

### 2.15 Mitochondria staining

For MitoTracker staining, macrophages were incubated with MitoTracker Green (100 nM) or MitoTracker Red CMXRos (500 nM) for 30 min at 37°C in the dark. Quantification of fluorescence intensity was performed using flow cytometry.

### 2.16 Human public dataset analysis

We obtained fecal metabolomics, clinical features, and colon tissue transcriptomic data from the publicly available Multi-omics Database of Inflammatory Bowel Disease (https://ibdmdb.org). Sample selection included adult UC patients, CD patients and non-IBD controls. Fecal LCA levels were originally measured by LC-MS (Thermo Fisher Scientific). Disease severity was analyzed according to the reported ESR or Calprotectin. ESR and fecal Calprotectin were obtained from the standardized clinical metadata provided by IBDMDB. For LCA-based grouping, patients were divided into high-LCA and low-LCA groups using the median fecal LCA concentration as the cut-off. Differential gene expression analysis was performed using DESeq2, and KEGG pathway enrichment was conducted using clusterProfiler. Immune cell proportions were estimated using CIBERSORT. Correlation between LCA and ESR was assessed by Spearman's rank correlation. All analyses were performed in R (version 4.5).

### 2.17 Statistics

Data were analyzed using GraphPad Prism software (version 9.0) and R software (version 4.5). All results are presented as the mean ± standard error of the mean (SEM). Differences between groups were assessed using one-way ANOVA and two-way ANOVA followed by Bonferroni *post-hoc* test for multiple comparisons. For comparisons between two groups, an unpaired Student's *t*-test was used. *P*-values less than 0.05 were considered statistically significant.

## 3 Results

### 3.1 5:2 IF accelerates the remission of DSS-induced chronic colitis

**Figure 1:**
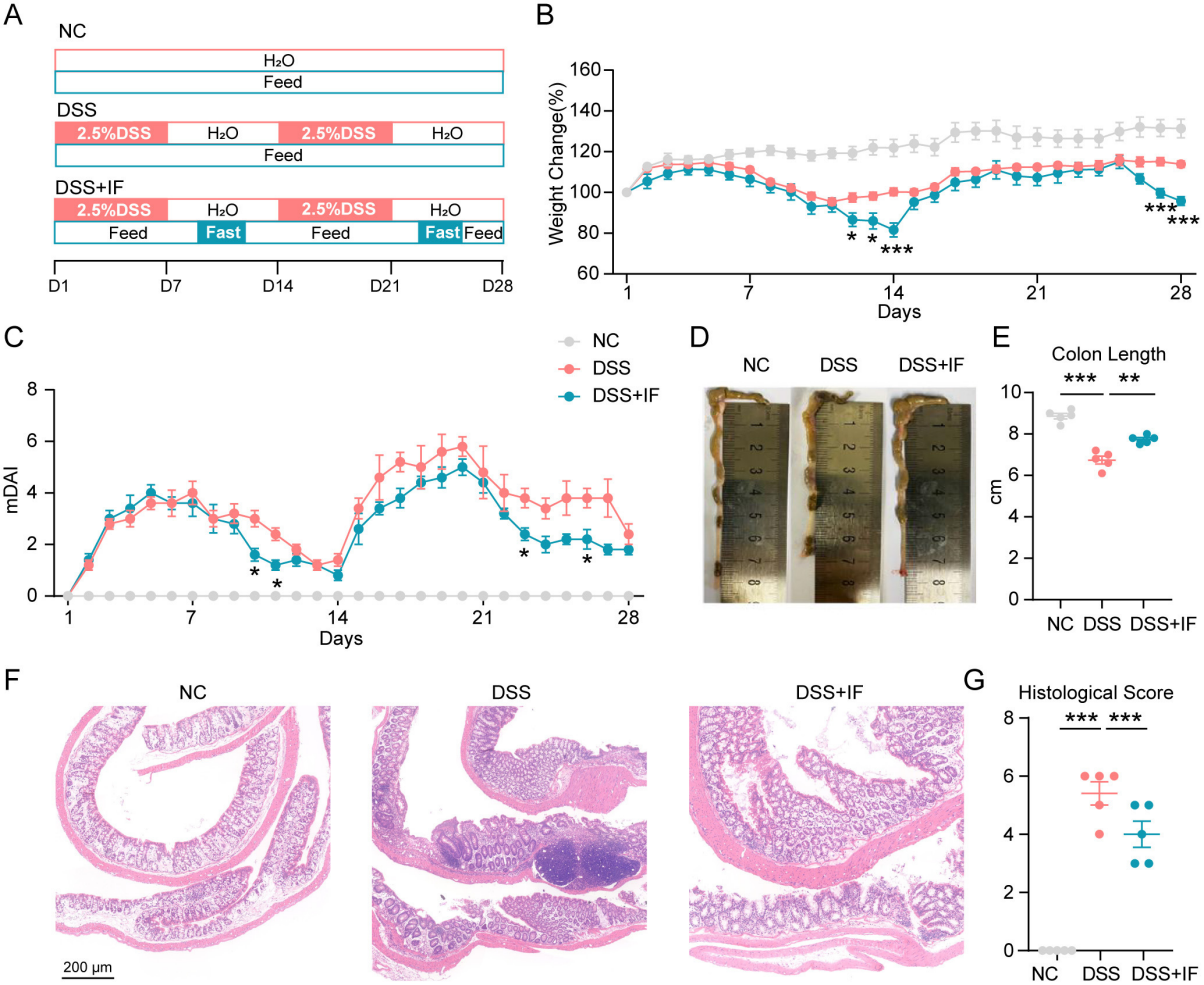
5:2 IF accelerates the remission of DSS-induced colitis **(A)** Schematic diagram. Weight change **(B)** and modified DAI **(C)** of mice in NC, DSS, DSS+IF group. *represents comparisons between DSS+IF group and DSS group. **(D)** Representative colon images and colon length **(E)** after treatment (*n* = 5). **(F)** Representative H&E-stained mouse colon sections. **(G)** Histological score of mouse colon sections. Data are mean ± SEM. Significance defined as *P < 0.05, **P < 0.01, ***P < 0.001.

To investigate the effect of IF on UC, two cycles of intervention were given over a span of 4 weeks to establish a chronic colitis model. Each cycle consisted of 6 or 7 days of 2.5% dextran sulfate sodium salt (DSS) drinking with *ad libitum* feeding, followed by 7 days of ddH_2_O with 5:2 IF, consisting of 5 days of *ad libitum* and 2 days of fasting in between (Figure 1A). The average amount of daily chow consumption was recorded for 14 days prior to the experiment. During fasting days, each animal was given 30% of their average normal chow intake. The effect of two cycles of 5:2 IF was evaluated based on modified disease activity index (mDAI), colon length and pathology. Comparison was made between normal control (NC) group, which received neither DSS nor IF intervention, a disease group administered with 2.5% DSS and fed regular chow *ad libitum* (DSS), and an intervention group given two cycles of DSS with 5:2 IF dietary restrictions (DSS+IF). Since implementation of IF greatly affected body weight (Figure 1B), mDAI was used to assess disease severity based on changes in stool consistency and blood in the stool. The DSS+IF group showed a decrease in mDAI score compared with DSS group during the recovery cycle (Figure 1C). A significant difference in colon length was also noted amongst the three groups (Figures 1D, E). The average colon length of the DSS group was 6.740 cm, while that of the DSS+IF group was 7.740 cm (*P* < 0.05). Hematoxylin and Eosin (H&E) staining showed different degrees of colonic inflammation. In comparison to the DSS group, DSS+IF group had a more intact epithelial layer and crypt structure, less infiltration of lymphocytes (Figures 1F, G), which confirmed that IF intervention significantly mitigated the severity of colitis in murine models. Moreover, H&E staining was performed on the spleen and liver of mice, the results indicated that there were no obvious pathological changes after receiving IF treatment (Supplementary Figures S1A, B).

### 3.2 IF promotes the accumulation of bile acids with an increase in LCA levels

**Figure 2:**
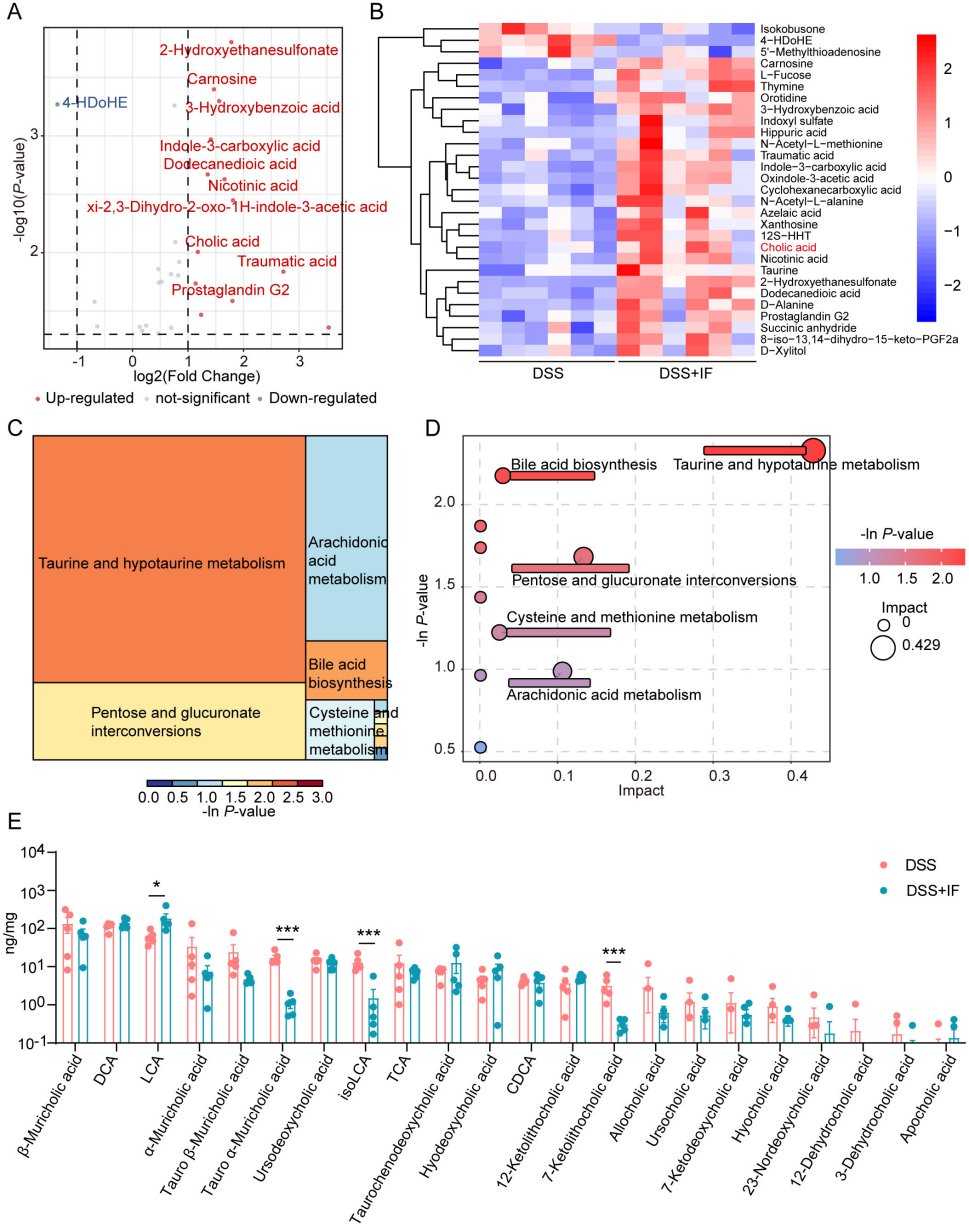
IF promotes the accumulation of bile acids with an increase in LCA levels **(A)** Volcano plot of differential colonic metabolites after IF compared to DSS (*n* = 6). **(B)** Heatmap showing the relative abundance of metabolites in the colon across the DSS, and DSS+IF groups (*n* = 6). **(C)** Tree map of the pathway enrichment analysis of differential metabolites. **(D)** Dot plot of the pathway enrichment analysis of differential metabolites. **(E)** Targeted metabolomic quantification of the colonic bile acid pool. The bar graph shows the specific alterations in individual bile acid subtypes in colonic tissues, ranked by their concentrations (*n* = 5). Data are mean ± SEM. Significance defined as *P < 0.05, ***P < 0.001.

To gain deeper insights into the molecular mechanisms underlying the protective effects of IF in colitis, we performed untargeted metabolomic profiling of murine colonic tissue. An initial screening revealed significant alterations in multiple metabolites. Notably, we observed a significant upregulation in cholic acid as well as nicotinic acid, 2-hydroxyethanesulfonate, traumatic acid and prostaglandin G2 and so on (Figures 2A, B). Differentially accumulated metabolites were enriched in metabolic pathways such as bile acid biosynthesis and taurine/hypotaurine metabolism (Figures 2C, D). Taurine and hypotaurine metabolism is closely linked to bile acid metabolism through taurine-conjugation of bile acids, which enhances bile acid solubility and bioactivity, and reciprocal regulation in the enterohepatic circulation (16). Given the emerging role of bile acids in metabolic regulation during fasting (17, 18), targeted metabolic profiling using mass spectrometry was employed to investigate specific alterations in colonic bile acid. The pooled concentration of total bile acids demonstrated a significant 3.1-fold elevation of LCA (*P* < 0.05), accompanied by a significant decrease in tauro-α-muricholic acid, isolithocholic acid (isoLCA), and 7-ketolithocholic acid (7-ketoLCA; Figure 2E). 7-ketoLCA is generated from chenodeoxycholic acid (CDCA) via 7α/β-hydroxysteroid dehydrogenase (7α/β-HSDH), whereas isoLCA is derived from LCA through microbial 3α-HSDH and 3β-HSDH (19, 20). Considering LCA is highly enriched in the colon and significantly elevated after IF treatment, LCA might tentatively contribute to the colitis-protective benefits conferred by IF.

### 3.3 Cholestyramine treatment reverses the protective effect of IF against DSS-induced colitis

**Figure 3:**
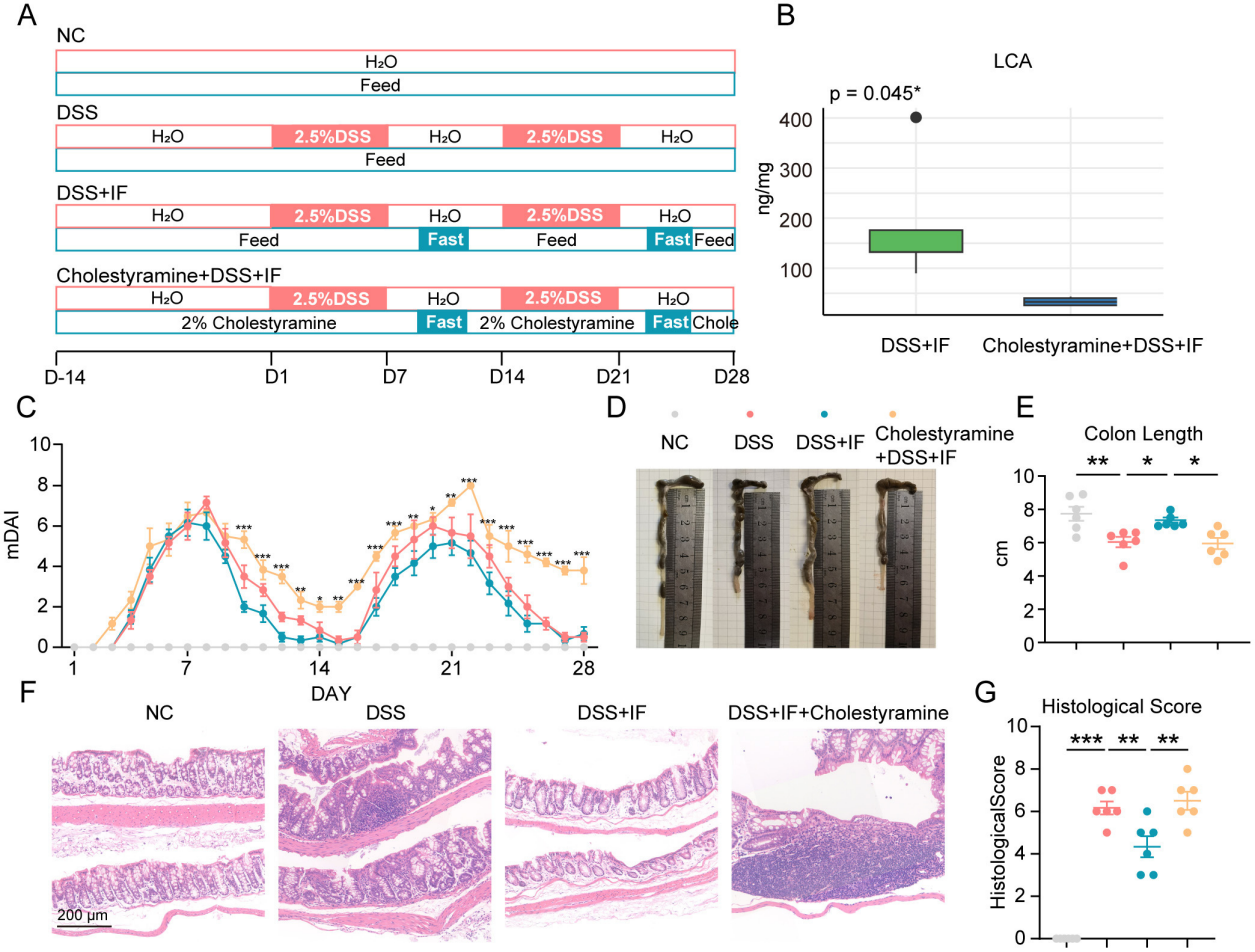
Cholestyramine treatment reverses the protective effect of IF against DSS-induced colitis **(A)** Schematic diagram. **(B)** Targeted metabolomic quantification of the concentration of LCA in colonic tissues. **(C)** mDAI of mice in NC, DSS, DSS+IF, and Cholestyramine+DSS+IF group, *represent comparisons between Cholestyramine+DSS+IF group and DSS+IF group (*n* = 6). **(D)** Representative colon images and colon length **(E)** of mice in NC, DSS, DSS+IF, and Cholestyramine+DSS+IF group (*n* = 6). **(F)** Representative H&E-stained mouse colon sections. **(G)** Histological score of mouse colon sections. Data are mean ± SEM. Significance defined as *P < 0.05, **P < 0.01, ***P < 0.001.

To assess the role of bile acids in IF-mediated colitis alleviation, including the IF-induced increase of LCA, we employed a cholestyramine intervention to eliminate bile acid reabsorption. Cholestyramine, a cationic exchange resin polymer can exert its effects by binding to bile acids in the intestine and blocking their enterohepatic reabsorption. Pretreatment with a diet containing 2% cholestyramine was given for 14 days prior to initiating two cycles of DSS and IF intervention (Figure 3A). Cholestyramine intervention significantly reduced LCA levels in the colon tissue samples (Figure 3B). Moreover, the ameliorating effects of IF on DSS-induced colitis were largely abolished in the cholestyramine group (Figure 3C). Colon length was further reverted and was comparable to that of the DSS group (Figures 3D, E). H&E staining revealed dense infiltration of lymphocytes and ulcer formation, with a lower histological score in the cholestyramine group compared to the IF group (Figures 3F, G). These results suggest that the colitis-alleviating effect of IF may involve bile acids, among which LCA could play a part.

### 3.4 LCA attenuates DSS-induced chronic colitis in mice

**Figure 4:**
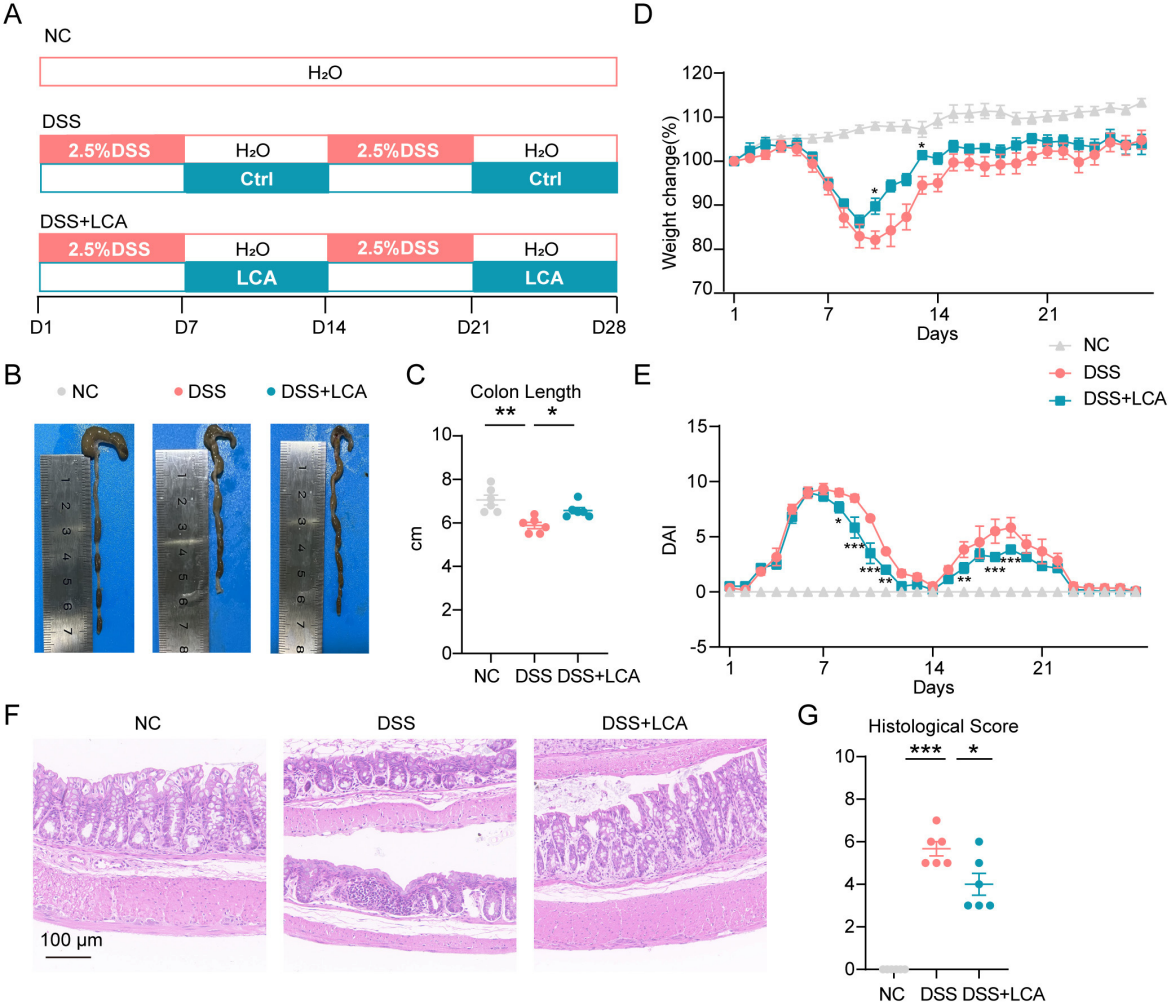
LCA attenuates DSS-induced chronic colitis in mice **(A)** Schematic diagram. **(B)** Representative colon images and colon length **(C)** after LCA treatment (*n* = 6). **(D)** Body weight change and disease activity index **(E)** of NC, DSS, DSS and LCA gavage group, *represent comparisons between DSS^+^LCA group and DSS group (*n* = 6). **(F)** Representative H&E-stained mouse colon sections. **(G)** Histological score of mouse colon sections. Data are mean ± SEM. Significance defined as *P < 0.05, **P < 0.01, ***P < 0.001.

To further elucidate the role of LCA in IF-mediated amelioration of DSS-induced chronic colitis, we conducted *in vivo* intervention experiments to determine whether LCA supplementation could replicate the protective effects of IF. LCA was administered via gavage daily during the recovery period (Figure 4A). The results demonstrated a significant improvement in the overall condition of mice in the DSS+LCA group compared to the DSS group. Specifically, mice treated with LCA exhibited increased body weight and colon length, along with reduced disease activity index (DAI) scores (Figures 4B–E). Histological analysis further supported these observations. H&E staining revealed the preservation of mucosal integrity in the DSS+LCA group, characterized by the absence of epithelial erosion, inflammatory cell infiltration, and crypt structural damage (Figures 4F, G). Taken together, supplementation with LCA significantly alleviated chronic colitis in mice, improving disease activity, reducing inflammation, and preserving mucosal integrity, suggesting gut-protective effects similar to those of IF.

### 3.5 LCA inhibits pro-inflammatory polarization of macrophage through enhancement of oxidative phosphorylation

**Figure 5:**
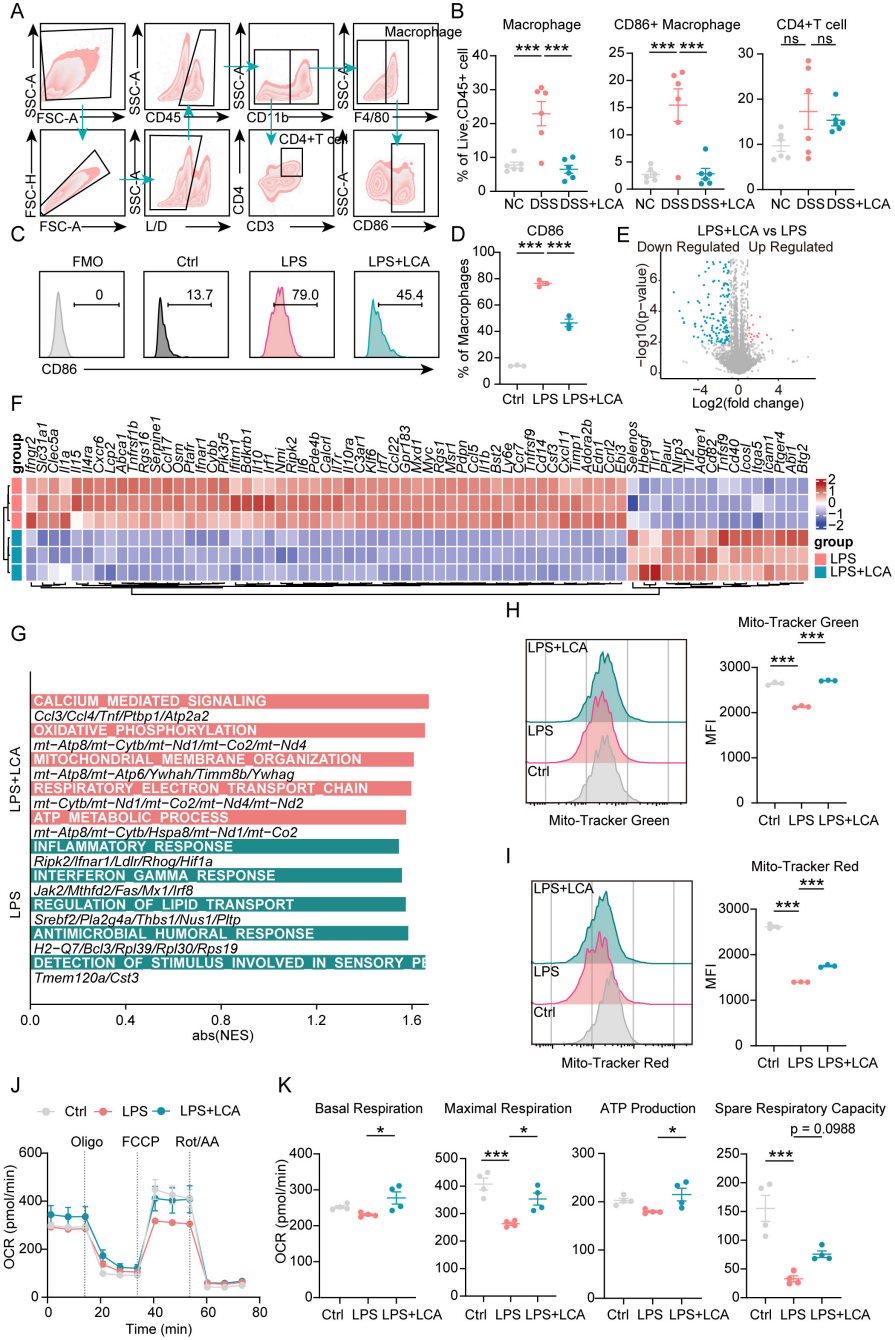
LCA inhibits pro-inflammatory polarization of macrophage through enhancement of oxidative phosphorylation **(A)** Gating strategy of immune cells in mouse colon lamina propria. **(B)** Proportion of immune cells in the colon lamina propria following LCA gavage (*n* = 6). **(C)** Histogram of CD86 expression in BMDMs following 100 ng/ml LPS and 30 μM LCA treatment (*n* = 3). **(D)** Percentage of CD86^+^ macrophages in cultured BMDMs (*n* = 3). **(E)** Volcano plot and **(F)** heatmap of DEGs following LCA and LPS treatment. **(G)** Up-regulated pathways of LPS and LCA treatment. **(H)** Histogram and mean fluorescence intensity (MFI) of Mito-Tracker Green staining in BMDMs (*n* = 3). **(I)** Histogram and MFI of Mito-Tracker Red staining in BMDMs (*n* = 3). **(J)** Oxygen Consumption Rate (OCR) of cultured BMDMs following treatment with LPS and LCA. **(K)** Measurement of basal respiration, maximal respiration, ATP production and spare respiratory capacity (*n* = 4). Data are mean ± SEM. Significance defined as *P < 0.05, ***P < 0.001, ns, not significant.

To further investigate the underlying mechanism, we extracted immune cells from the colonic lamina propria for flow cytometry analysis. The results showed a significant reduction in the proportion of macrophages as well as CD86^+^ macrophages in the LCA treated group compared to DSS treatment alone, implying that LCA exerts its therapeutic effects by modulating macrophage in the inflamed intestinal environment (Figure 5A, B). Although a decreasing trend was also observed in the proportion of CD4^+^ T cells, the changes did not reach statistical significance. To establish the relationship between LCA and macrophages, we first cultured bone marrow-derived macrophages (BMDMs) *in vitro* and induced their pro-inflammatory polarization using lipopolysaccharide (LPS), followed by the addition of LCA (30 μM) for treatment. The results of flow cytometry showed that macrophages polarized to pro-inflammatory type under LPS stimulation, with a significant increase in CD86 and iNOS expression. The addition of LCA significantly inhibited LPS-induced pro-inflammatory macrophage formation, as evidenced by a decrease in CD86 and iNOS expression (Figures 5C, D, Supplementary Figures S3A–C). To assess the effect of LCA on macrophage inflammation, we performed gene expression profiling of BMDMs. The results revealed that LCA intervention led to the upregulation of 23 genes and downregulation of 229 genes relative to LPS stimulation (Figure 5E). Heatmap based clustering analysis of these differentially expressed genes demonstrated LCA treatment markedly suppressed pro-inflammatory gene signature (*Il1a, Il1b, Il6*) and chemokines (e.g., *Cxcr6, Ccl22, Ccl5*) and inflammatory signaling receptors (*Tnfrsf1b, Ifngr2*) (Figure 5F). To further assess the regulatory effect of LCA on anti-inflammatory macrophage polarization, BMDMs were stimulated with IL-4 to induce anti-inflammatory polarization. Flow cytometry analysis showed that LCA intervention led to an increasing trend in CD206 compared to the IL-4 alone group, although this difference did not reach statistical significance (*P* = 0.0610) (Supplementary Figures S3D, E). Furthermore, quantitative PCR analysis revealed that LCA treatment significantly upregulated the mRNA expression levels of *Il10* and *Tgfb1* (Supplementary Figures S3F, G). These observations collectively indicate that LCA intervention exerts a significant inhibitory effect on macrophage-mediated inflammatory responses, as evidenced by the downregulation of key pro-inflammatory cytokines, the upregulation of anti-inflammatory cytokines, and the modulation of macrophage polarization-associated molecules.

To elucidate the potential mechanism underlying LCA mediated macrophage regulation, we performed a comparative analysis of differentially expressed genes (DEGs) in macrophages treated with LCA vs. LPS. Transcriptomic profiling revealed significant upregulation of calcium mediated signal, oxidative phosphorylation pathway and ATP metabolic process following LCA intervention (Figure 5G). Mitochondrial oxidative phosphorylation orchestrates metabolic reprogramming to drive macrophage polarization by modulating energy supply and redox signaling, thereby influencing the balance between pro-inflammatory and anti-inflammatory phenotypes (21). To determine whether LCA affects cellular oxidative phosphorylation levels, we first evaluated mitochondrial mass in macrophages using MitoTracker Green staining. LPS intervention led to a notable decrease in MitoTracker Green fluorescence intensity, while LCA treatment reversed this LPS induced reduction (Figure 5H), suggesting that LCA promotes an increase in mitochondrial mass. Furthermore, mitochondrial membrane potential was assessed via MitoTracker Red staining. LPS intervention caused a significant downregulation of the mitochondrial membrane potential, which was remarkably reversed by LCA (Figure 5I). To further characterize cellular oxidative phosphorylation, we performed Seahorse assays and observed that LCA enhanced basal respiration, maximal respiratory capacity and ATP production under LPS treatment (Figures 5J, K). Altogether, these findings demonstrate that LCA enhances mitochondrial membrane potential and improves oxidative phosphorylation capacity in macrophages, which underlies its inhibitory effects on the pro-inflammatory function of macrophages.

Given that LCA is a well-characterized FXR antagonist and TGR5 agonist (22, 23), we pre-treated BMDMs with GW4064 (an FXR agonist, 10 μM) or SBI-115 (a TGR5 antagonist, 1 μM) prior to stimulation with LPS in the presence or absence of LCA to investigate whether the effects of LCA on macrophage polarization are mediated by canonical bile acid receptors. Flow cytometry analysis showed that LCA failed to reduce the proportion of CD86^+^ macrophages following GW4064 intervention (Supplementary Figure S4A, B). However, following SBI-115 treatment LCA still efficiently reduced the proportion of CD86^+^ macrophages (Supplementary Figures S4A, B). Seahorse analysis was further performed to verify whether GW4064 could also regulate LCA-mediated enhancement of oxidative phosphorylation in macrophages. The results showed that LCA still promoted basal respiration, maximal respiration, and ATP production upon GW4064 treatment, but to a lesser extent. Compared with the LCA-alone group, additional GW4064 intervention led to significant reductions in basal respiration, maximal respiration and ATP production (Supplementary Figures S4C, D). Collectively, these results suggest that GW4064 blocks the LCA-induced reduction in CD86^+^ macrophages and partially blunts LCA-mediated enhancement of oxidative phosphorylation. These findings indicate that FXR is partially involved in LCA-driven regulation of macrophage polarization and mitochondrial oxidative phosphorylation.

### 3.6 IF elevates LCA levels through modulating gut microbiota

**Figure 6:**
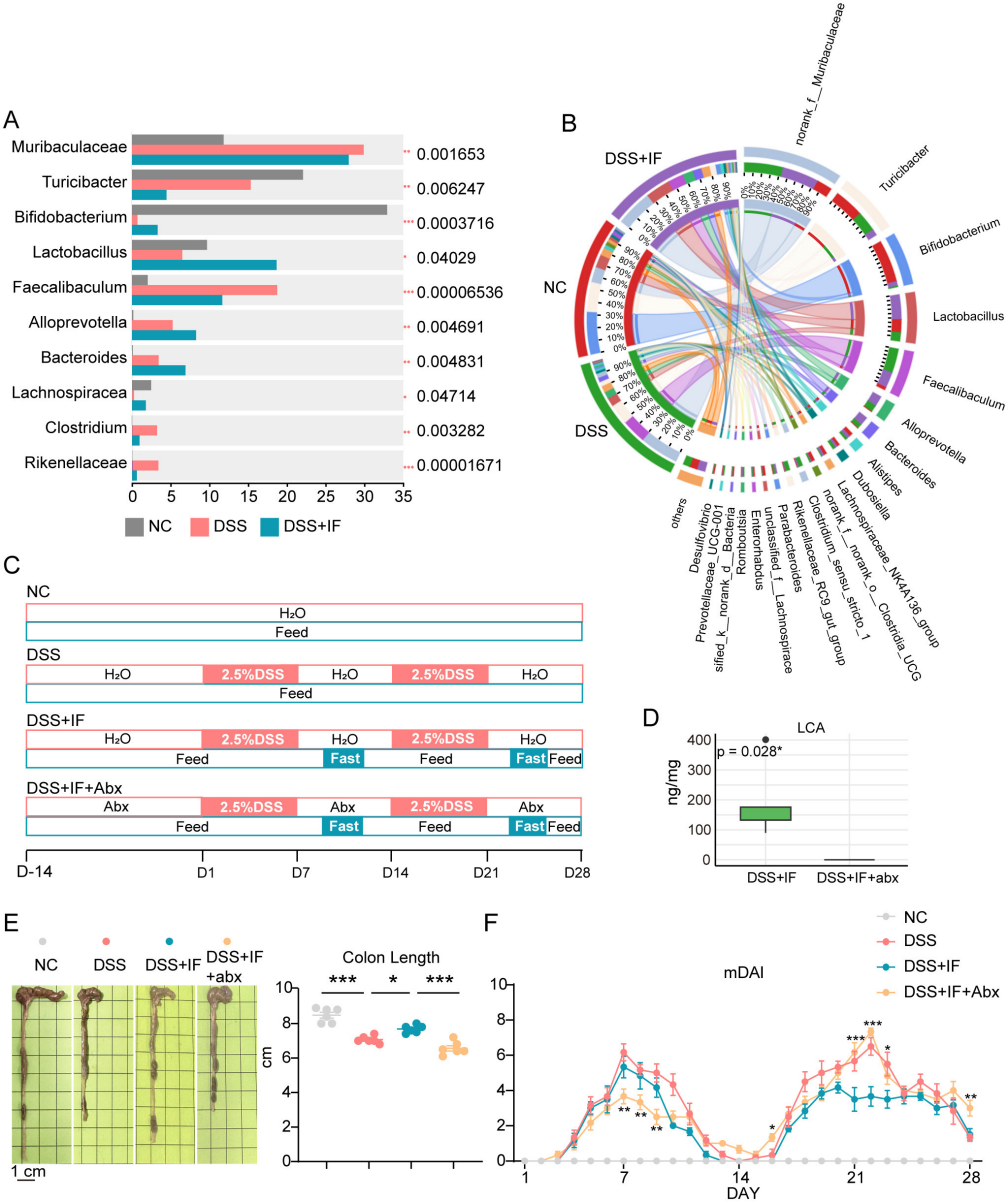
IF elevates LCA levels through modulating gut microbiota **(A)** Bar plot of relative abundance of representative differentially composed microbiota (*n* = 6). **(B)** Chord diagram of relative abundance of representative microbiota. **(C)** Schematic diagram. **(D)** Targeted metabolomic quantification of the concentration of LCA in colonic tissues. **(E)** Representative colon images and colon length after *Abx* treatment (*n* = 6). **(F)** Modified DAI of mice in NC, DSS, DSS+IF group, DSS+IF+*Abx* group, *represent comparisons between DSS+IF+*Abx* group and DSS+IF group (*n* = 6). Data are mean ± SEM. Significance defined as *P < 0.05, **P < 0.01, ***P < 0.001.

It has been reported that the gut microbiota participates in bile acid metabolism through enzymatic modification of bile acid structures (24). To further elucidate how IF modulates bile acid metabolism and upregulated LCA, stool samples from mice were collected after dietary intervention. The relative abundance of different genera differed among three groups. Specifically, *Bifidobacterium, Lactobacillus* and *Bacteroides* were significantly increased after IF intervention compared to DSS group, while *Turicibacter, Faecalibaculum*, and *Clostridium* were downregulated by IF (Figures 6A, B). Furthermore, a subsequent correlation analysis between colonic bile acids and microbiota revealed a robust correlation between them, particularly *Bifidobacterium* species (Supplementary Figure S2). To further investigate the role of the gut microbiota in mediating IF-induced LCA elevation and its subsequent protective effects against colitis, the mice were pretreated with an antibiotics (*Abx)* cocktail 14 days prior to IF intervention (Figure 6C). Notably, *Abx* treatment significantly suppressed colonic LCA levels (Figure 6D), which demonstrates that the upregulation of LCA by IF is mediated by the remodeling of the gut microbiota. Compared with DSS+IF group, the *Abx* group had an increased mDAI score and a decrease in colon length (Figures 6E, F). To explore the microbial functional basis underlying IF-mediated bile acid metabolic remodeling, we analyzed the gut microbial function related to secondary bile acid biosynthesis using PICRUSt2. The pathway abundance of secondary bile acid biosynthesis was increased in the DSS+IF group compared with the DSS group (Supplementary Figure S5A). Correlation analysis revealed that the relative abundance of *Bifidobacterium* was significantly and positively correlated with the activity of the secondary bile acid biosynthesis pathway, whereas no significant correlations were observed for *Negativibacillus* or *Dubosiella* (Supplementary Figure S5B). Furthermore, predictive analysis of key bile acid-metabolizing enzymes showed that, relative to the DSS group, the DSS+IF group had increased predicted abundance of *baiB* and *baiH* and decreased predicted abundance of *baiN*, with no significant differences in the predicted abundance of *3*α*-HSDH, BSH*, and *hdhA* among the three groups (Supplementary Figure S5C). Altogether, these results indicate that the gut microbiota contributes to LCA production and mediates the protective effects of IF against colitis.

### 3.7 High levels of LCA correlate with remission of UC patients with enhanced mitochondrial activity and reduced pro-inflammatory macrophage

**Figure 7:**
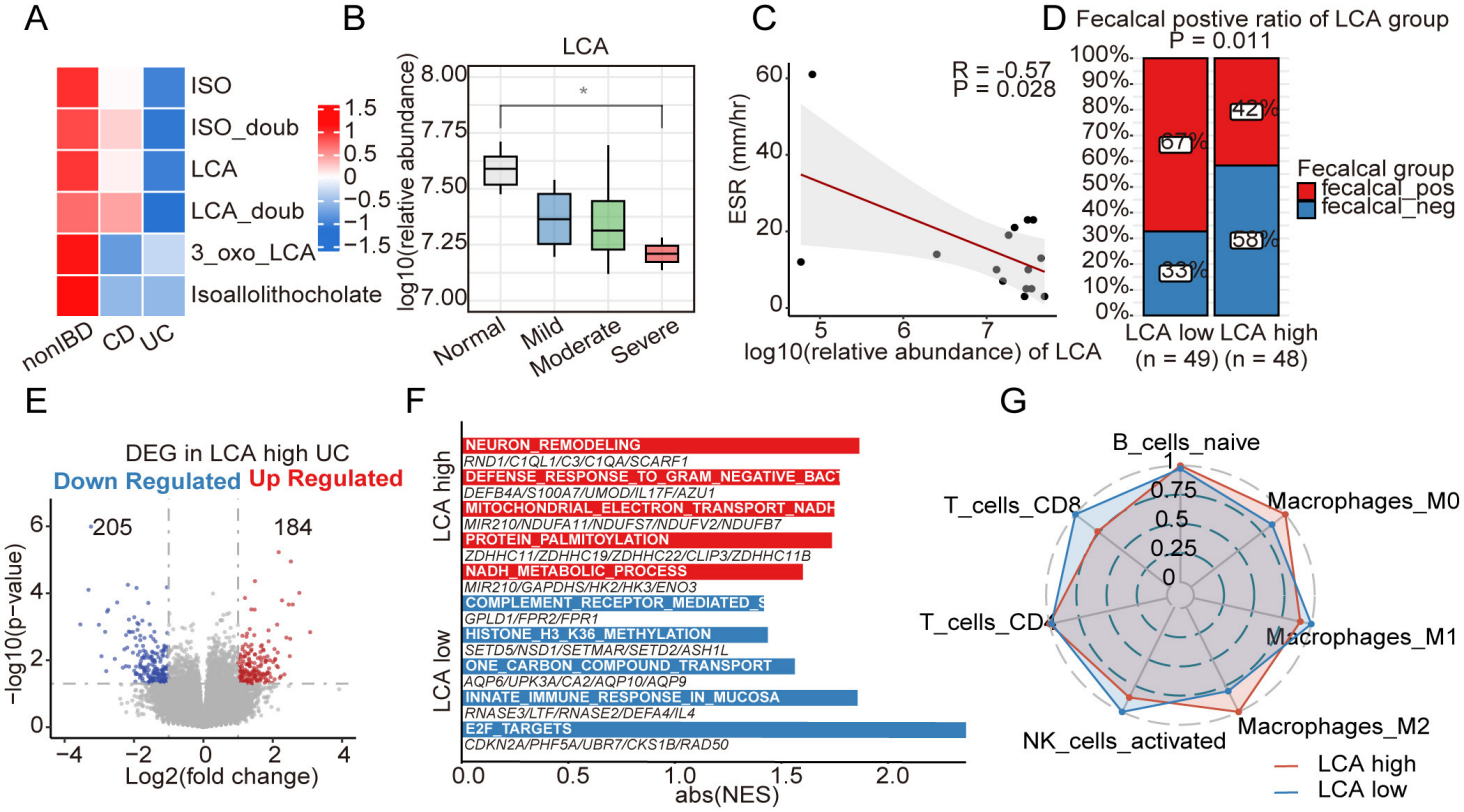
High levels of LCA correlate with remission of UC patients with enhanced mitochondrial activity and reduced pro-inflammatory macrophage **(A)** Heatmap of LCA isoform concentrations in non-IBD (individuals not diagnosed with CD or UC), CD, and UC patients. **(B)** LCA abundance in stool sample from patients with different disease severity. **(C)** Correlation between fecal LCA level and plasma ESR level. **(D)** Patients were divided into high and low groups based on their LCA levels compared to the median LCA level, and the fecal calprotectin positive ratio was compared between the two groups. **(E)** Volcano plots of DEGs in patients with high LCA levels compared to patients with low LCA levels. **(F)** Up-regulated pathways in patients with high and low LCA levels. **(G)** Proportion of immune cells in patients with high (red) and low LCA (cyan) levels, calculated using CIBERSORT. Data are mean ± SEM. Significance defined as *P < 0.05.

As demonstrated in our *in vivo* and *in vitro* experiments, LCA can act through the metabolic reprogramming in intestinal macrophages to suppress inflammatory responses, thereby alleviating UC. To explore whether similar associations exist in human UC patients, we conducted an integrated analysis of public datasets (25). The results revealed that the level of stool LCA was significantly decreased in UC patients, and various isoforms of LCA also showed a downward trend (Figure 7A). Additionally, we found that the reduction in LCA level was associated with the aggravation of disease severity (Figure 7B). We further analyzed the relationship between LCA level and erythrocyte sedimentation rate (ESR), which is an important indicator for evaluating the severity of UC. The concentration of LCA was significantly negatively correlated with the level of ESR (Figure 7C), suggesting that an increase in LCA is associated with remission of UC. Furthermore, we divided the patients into low LCA group and high LCA group based on their fecal LCA levels. The results showed that higher proportion of patients in the high LCA group had more negative calprotectin levels (Figure 7D), which is primarily produced by neutrophils and released during inflammation (26), serving as a key biomarker for assessing intestinal inflammatory activity. We further analyzed the transcriptomic differences in colonic tissues between patients with high LCA and low LCA levels. The results showed that there were 184 upregulated genes and 205 downregulated genes in the LCA high group compared to LCA low group (Figure 7E). Subsequent KEGG pathway analysis of these differentially expressed genes revealed an enrichment of the mitochondrial electron transport chain pathway in the LCA high group, while the activation of innate immune response was observed in the LCA low group (Figure 7F). Additionally, we performed CIBERSORT scoring in both groups and found that the LCA high group exhibited a lower enrichment of M1 type macrophages and higher enrichment of M2 type immune cells (Figure 7G). Conclusively, high level of LCA indicates reduced disease severity in patients with UC, which is accompanied by a decreased infiltration of pro-inflammatory macrophages and an elevated level of oxidative phosphorylation.

## 4 Discussion

Our study provides compelling evidence that IF, particularly the 5:2 regimen, significantly alleviates colitis symptoms in DSS-induced mouse models. This effect is mediated through the modulation of bile acid metabolism and immune cell reprogramming, especially macrophage dynamics. Collectively, these findings provide a mechanistic basis for considering dietary interventions in future studies on UC.

Dietary interventions have gained traction as a safe and effective strategy for various diseases, including metabolic disorders, cancer, and autoimmune diseases (27, 28). Previous studies have shown that fasting mimicking diet ameliorates colitis in murine models (29, 30). In line with these reports, our study demonstrated that two consecutive cycles of the 5:2 fasting regimen significantly reduced disease severity, as reflected by a decrease in the mDAI score, an increase in colon length, and improved histological parameters.

Notably, prior work has found that IF can affect the content of intestinal fecal metabolites in mice with colitis, including reduced α-muricholic acid (αMCA) and CA, along with elevated LCA and hyodeoxycholic acid (HDCA) (18). Consistent with these observations, our results showed that intestinal tissues of DSS-induced colitis mice after IF intervention shared a similar increase in LCA levels and a decrease in αMCA levels. However, in our study, the intestinal CA level was increased, while the content of HDCA in intestinal tissues remained unchanged. Furthermore, although fecal 7-ketoLCA showed no significant difference in previous studies, our study found that IF intervention significantly upregulated the level of 7-ketoLCA in the intestinal tissues. Critically, intestinal immune cells interact directly with the local metabolic microenvironment rather than with fecal metabolites, which do not fully recapitulate the *in situ* metabolic milieu experienced by mucosal immune cells. By directly measuring bile acid metabolites in intestinal tissues, our approach more accurately reflects metabolic changes within local lesion sites.

One of the key metabolic shifts induced by IF in our study was the alteration of bile acid profiles, particularly the upregulation of LCA. Bile acids have traditionally been recognized for their role in lipid metabolism, but recent studies have highlighted their immunomodulatory effects, particularly through interactions with nuclear receptors such as FXR and TGR5 (31). Additionally, LCA has been reported to directly bind TULP3 and activate AMPK (32). LCA, specifically, has been identified to alleviate inflammation in an acute DSS-induced injury repair model by suppressing the epithelial TLR4/NF-κB/NLRP3 pathway (33), consistent with the anti-inflammatory actions of LCA observed in our study. However, unlike previous studies using acute DSS models, we employed a chronic DSS-induced colitis paradigm that more closely recapitulates human UC, which is typified by persistent mucosal inflammation and barrier impairment. Using both *in vitro* and *in vivo* approaches, we found that direct LCA intervention effectively inhibited pro-inflammatory macrophage polarization, as evidenced by significantly reduced expression of CD86 and pro-inflammatory cytokines. Given that LCA serves as an FXR antagonist (22), we found that pre-treatment with GW4064, an FXR agonist, blocked the LCA-induced reduction of pro-inflammatory macrophages, suggesting that FXR signaling may be involved in the regulatory effects of LCA on macrophage polarization.

The pathogenesis of UC is multifaceted, involving a combination of genetic, environmental, microbial, and immune factors. Macrophages play a central role in maintaining intestinal homeostasis and shaping immune responses in UC (34). Under inflammatory conditions, macrophages exhibit a metabolic shift characterized by excessive activation and infiltration, ultimately exacerbating intestinal damage (35, 36). During active disease states, macrophages predominantly exhibit a pro-inflammatory phenotype, contributing to UC progression through the secretion of cytokines such as IL-6 and IL-1β. Our study revealed that IF significantly alters macrophage polarization, promoting a decrease of the pro-inflammatory phenotype. This alteration was associated with increased LCA levels, suggesting that bile acid metabolism plays a role in directing macrophage fate. Interestingly, the relationship between bile acids and macrophage polarization is complex. While LCA inhibits pro-inflammatory polarization, other bile acids, such as DCA and CDCA, have been implicated in pro-inflammatory pathways. High-fat diet studies have demonstrated that DCA promotes M1 polarization via the TLR2-M2-mAChR/Src pathway, exacerbating inflammation (37). Similarly, CDCA has been shown to inhibit M2 polarization by increasing mitochondrial superoxide accumulation through the ROS/p38 MAPK/DGAT1 pathway, thereby suppressing immune regulation (38).

Beyond macrophages, Th17 cells also participate in intestinal inflammation, and their differentiation can be modulated by gut microbiota-derived metabolites (39). Previous work reported that isoLCA, but not LCA itself, inhibits Th17 differentiation, and LCA gavage in germ-free mice does not affect intestinal Th17 cells (40). In our study, LCA gavage led to a non-significant reduction in CD4^+^ T cells, which might be attributed to downstream metabolites such as isoLCA rather than LCA directly. As we did not specifically analyze Th17 subsets, we cannot exclude their potential involvement in IF- or LCA-mediated colitis alleviation, and this warrants further investigation.

Mitochondrial oxidative respiration plays a pivotal role in regulating macrophage polarization. Emerging evidence indicates that mitochondrial oxidative phosphorylation serves as a key metabolic checkpoint in this context, while pro-inflammatory polarization is often associated with a shift toward glycolytic metabolism to support rapid energy production and the synthesis of pro-inflammatory mediators (41–43). The ability of LCA to enhance mitochondrial volume, preserve membrane potential, and improve oxidative phosphorylation in macrophages suggests the involvement of the underlying signaling pathways that may orchestrate these mitochondrial adaptations.

We acknowledge several limitations. First, the present evidence is derived primarily from murine models and correlative human datasets. The sample sizes in our animal experiments are modest, and the public human dataset lacks sufficient power for definitive causal conclusion. Second, while our antibiotic experiments support a role for the microbiota, more rigorous controls are needed to strengthen causal inference. Third, the 5:2 IF regimen involves both fasting and overall caloric restriction, further studies including a matched continuous energy restriction control group are needed to dissociate the specific effects of the fasting period *per se*.

## 5 Conclusion

Our findings demonstrate that IF, particularly the 5:2 regimen, effectively alleviates colitis symptoms in a chronic DSS mouse model through a multifaceted mechanism involving bile acid metabolism, immune regulation and metabolic reprogramming. The identification of LCA as a contributing metabolite in this process provides novel insights into how fasting exerts its beneficial effects. Specifically, LCA inhibits pro-inflammatory macrophage polarization by boosting oxidative respiration, contributing to a reduction in intestinal inflammation.

By bridging dietary intervention, metabolic remodeling, and immune regulation, this study offers a mechanistic basis for further exploring IF-related strategies in preclinical research on ulcerative colitis. While IF represents a promising experimental approach, additional studies are required to evaluate its safety, feasibility, and efficacy in future translational settings.

## Data Availability

The raw sequencing data generated in this study have been deposited in the NCBI Sequence Read Archive (SRA) under the BioProject accession number PRJNA1435820.
