# Supplementary material for: Intermittent fasting alleviates ulcerative colitis via lithocholic acid-mediated macrophage reprogramming
Source: Front Nutr. 2026 May 25;13:1841890. doi: 10.3389/fnut.2026.1841890 (PMC13243278; doi:10.3389/fnut.2026.1841890)
Supplement: Supplementary file 1 [file Supplementary_file_1.docx]

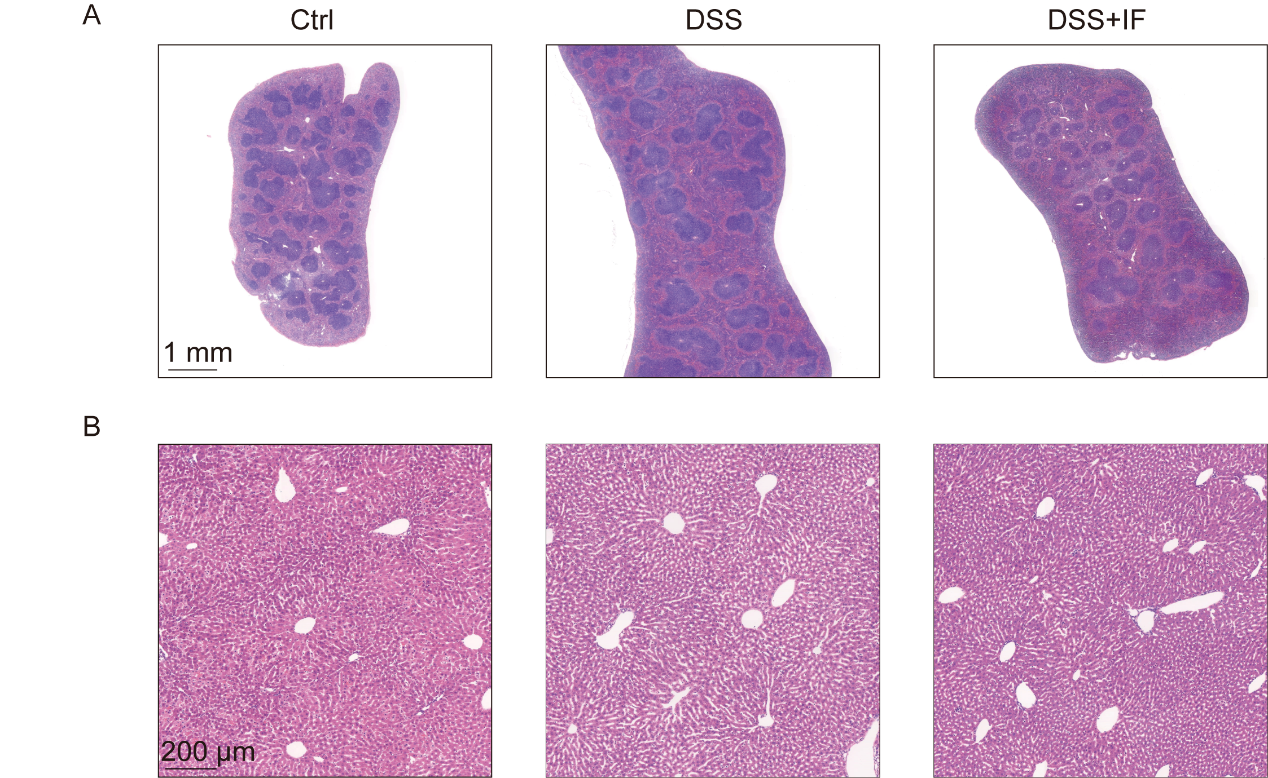


**Supplementary Figure 1.** H&E images of spleen (A) and liver (B) after IF treatment.


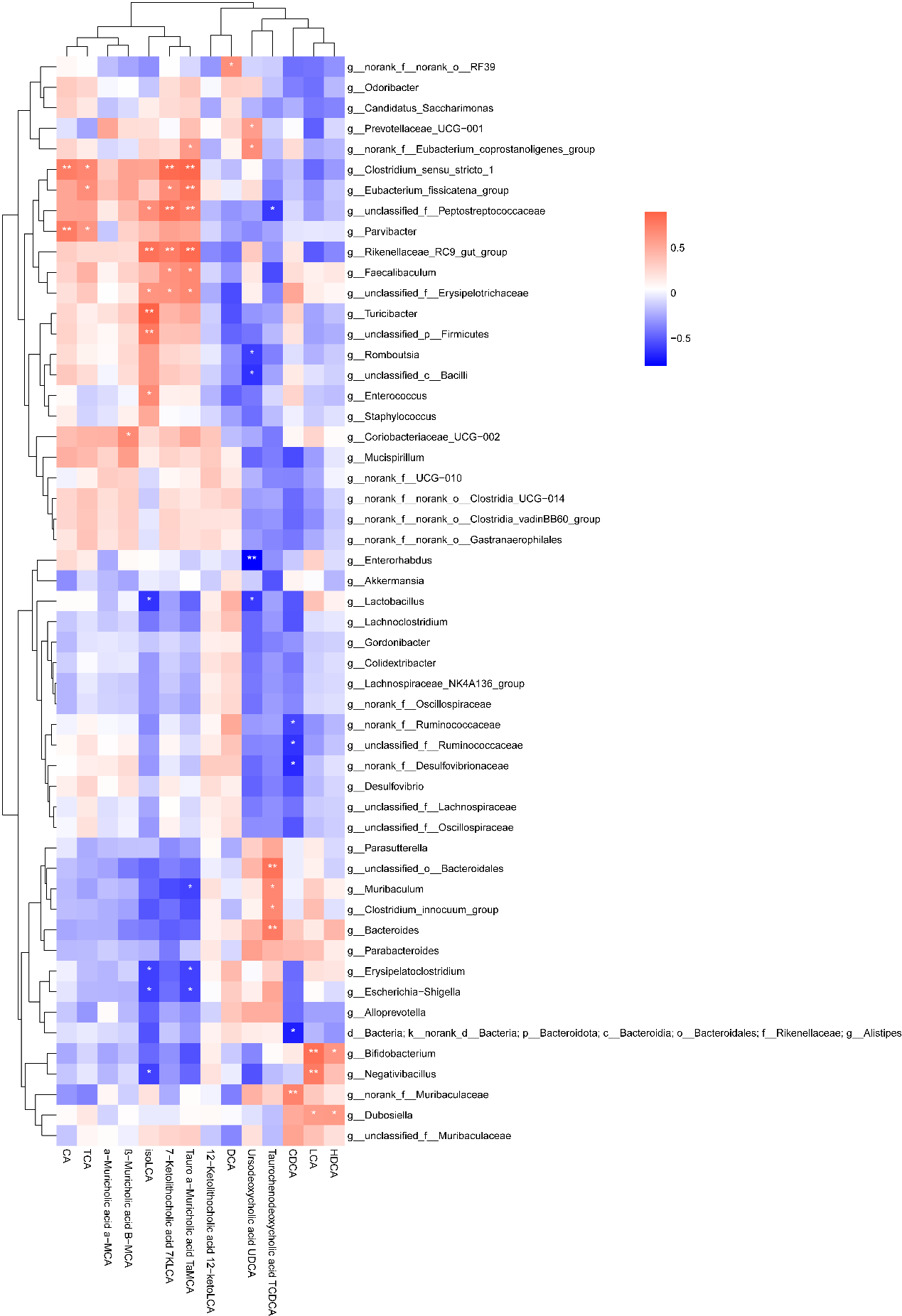


**Supplementary Figure 2.** Heatmap of correlation analysis between gut microbiota and colonic metabolites


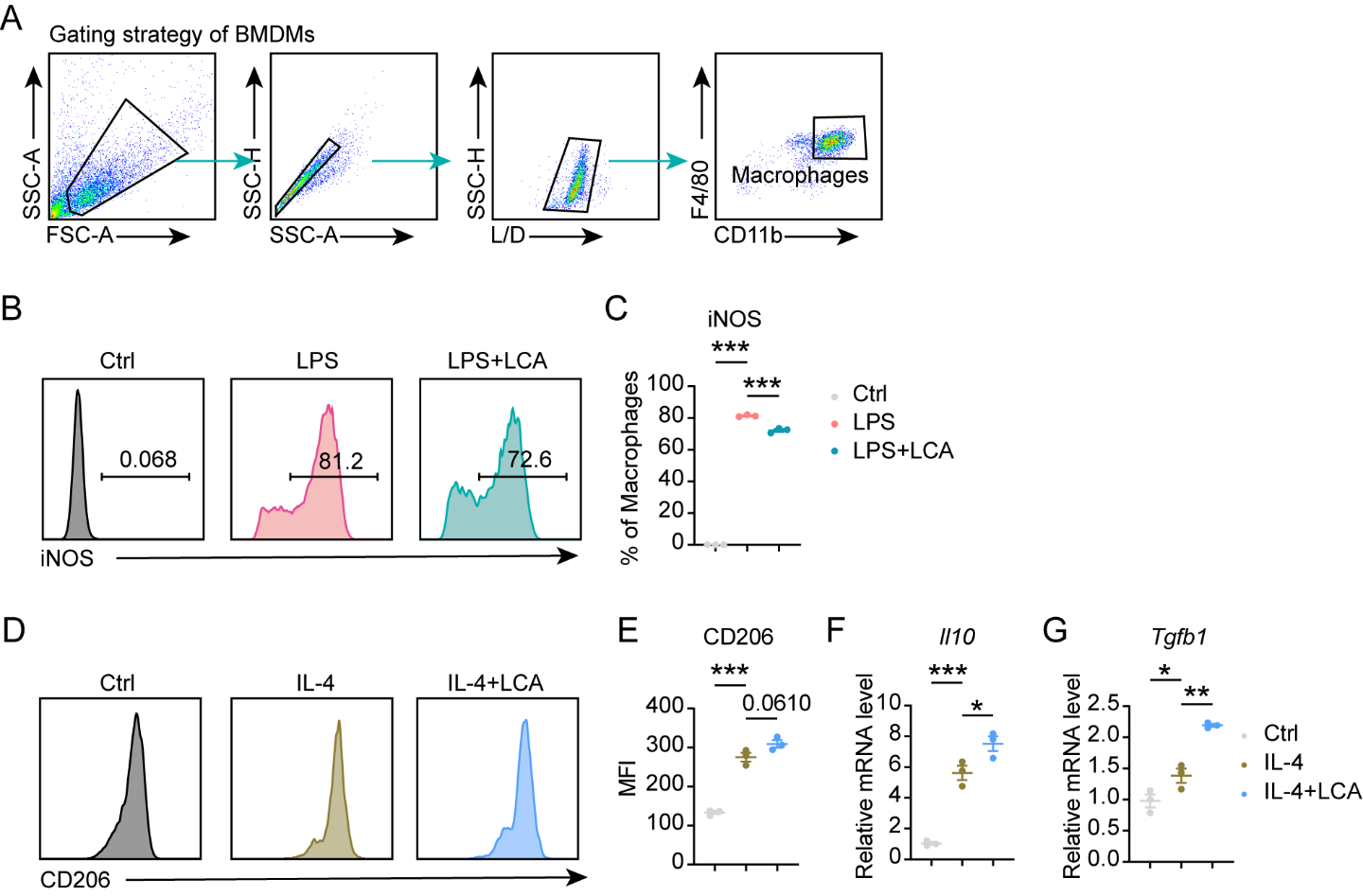
**Supplementary Figure 3.** Effect of LCA on macrophage polarization

**(A)** Gating strategy of BMDMs. **(B)** Histogram of iNOS expression in BMDMs following 100 ng/mL LPS and 30 μM LCA treatment. **(C)** Percentage of iNOS^+^ macrophages in cultured BMDMs (n = 3). **(D)** Histogram of CD206 expression in BMDMs following 20 ng/mL IL-4 and 30 μM LCA treatment (n = 3). **(E)** MFI of CD206 expression in cultured BMDMs (n = 3). **(F)** Relative mRNA level of *Il10* following LCA treatment (n = 3). **(G)** Relative mRNA level of *Tgfb1* following LCA treatment (n = 3). Data are mean ± SEM. Significance defined as *P < 0.05, **P < 0.01, ***P < 0.001.


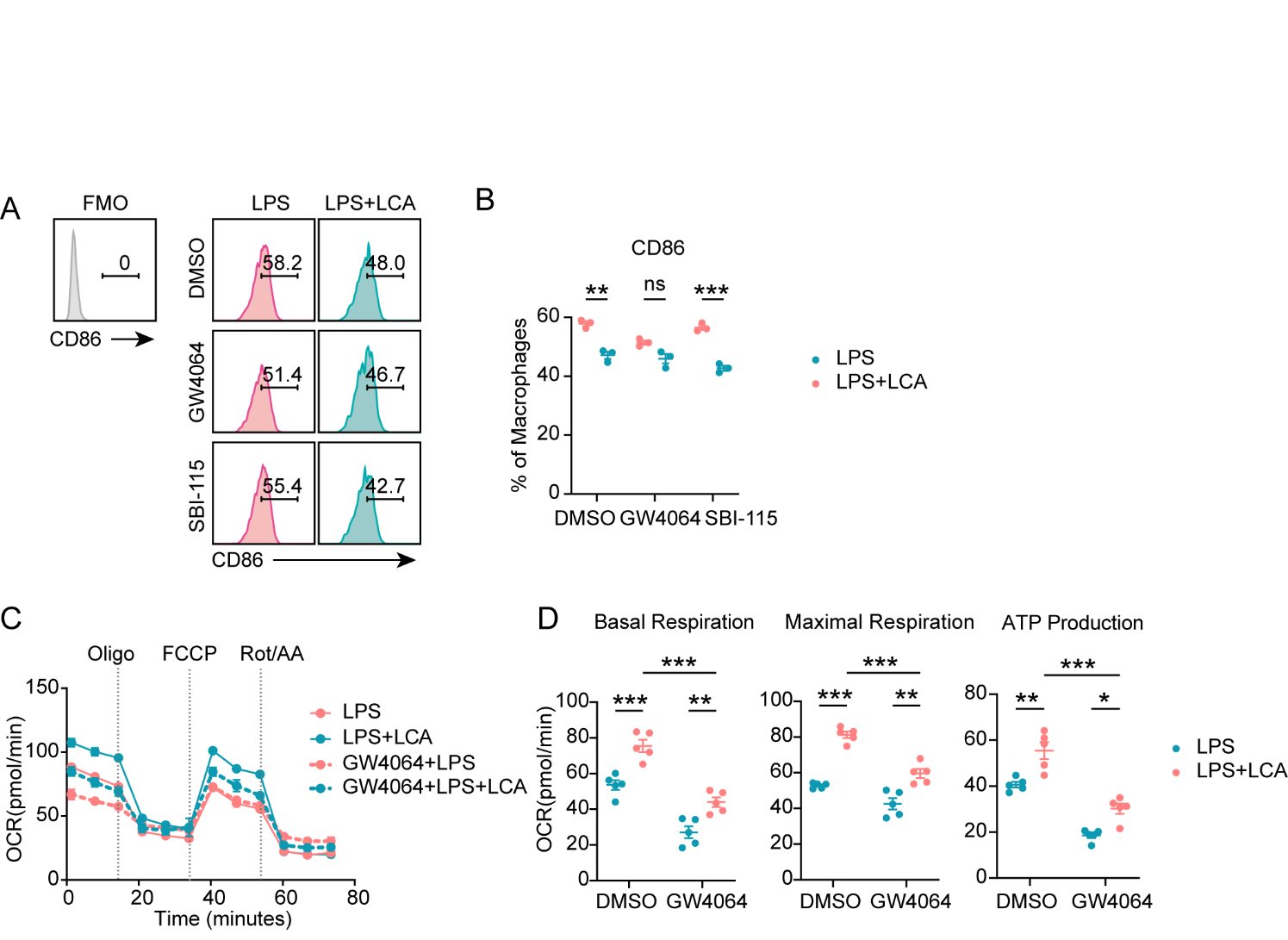


**Supplementary Figure 4.** FXR partially mediates LCA‑induced regulation of macrophage polarization and mitochondrial oxidative phosphorylation

**(A)** Histogram of CD86 expression in BMDMs. **(B)** Percentage of CD86^+^ macrophages in BMDMs (n = 3). **(C)** Oxygen Consumption Rate (OCR) of BMDMs following treatment with LPS, LCA and GW4064. **(D)** Measurement of basal respiration, maximal respiration and ATP production (n = 5). Data are mean ± SEM. Significance defined as *P < 0.05, **P < 0.01, ***P < 0.001, ns: not significant.


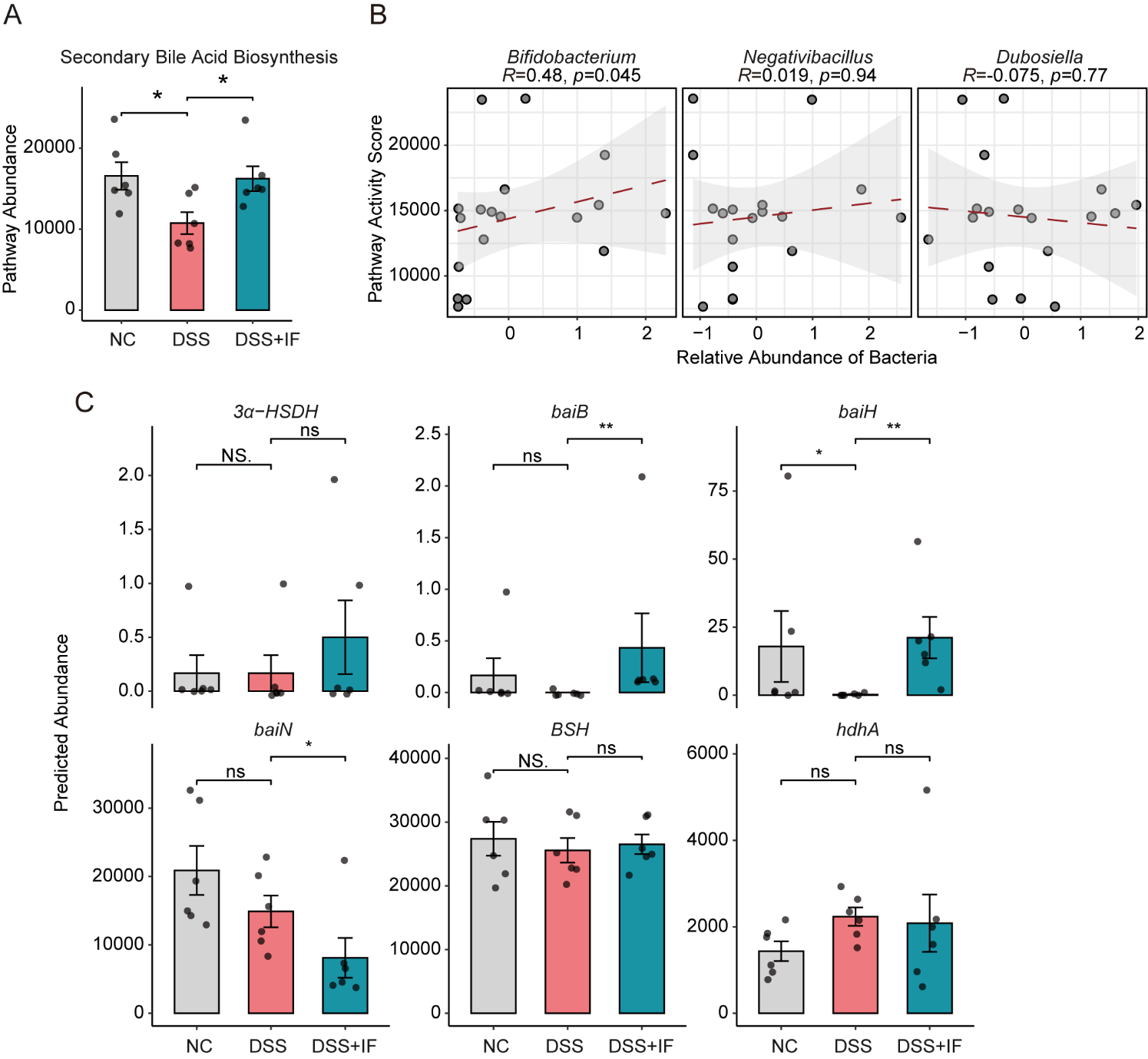


**Supplementary Figure 5.** Gut microbiota functional analysis related to bile acid metabolism following IF treatment

**(A)** Pathway abundance of secondary bile acid biosynthesis. **(B)** Correlation analysis between gut microbiota and pathways involved in secondary bile acid biosynthesis. **(C)** Enrichment analysis of predicted functional genes linked to bile acid synthesis enzymes among NC, DSS and DSS+IF groups. Data are mean ± SEM. Significance defined as *P < 0.05, **P < 0.01, ns: not significant.
