# Supplementary figures and images for: Intermittent fasting alleviates ulcerative colitis via lithocholic acid-mediated macrophage reprogramming
Source: Front Nutr. 2026 May 25;13:1841890. doi: 10.3389/fnut.2026.1841890 (PMC13243278; doi:10.3389/fnut.2026.1841890)

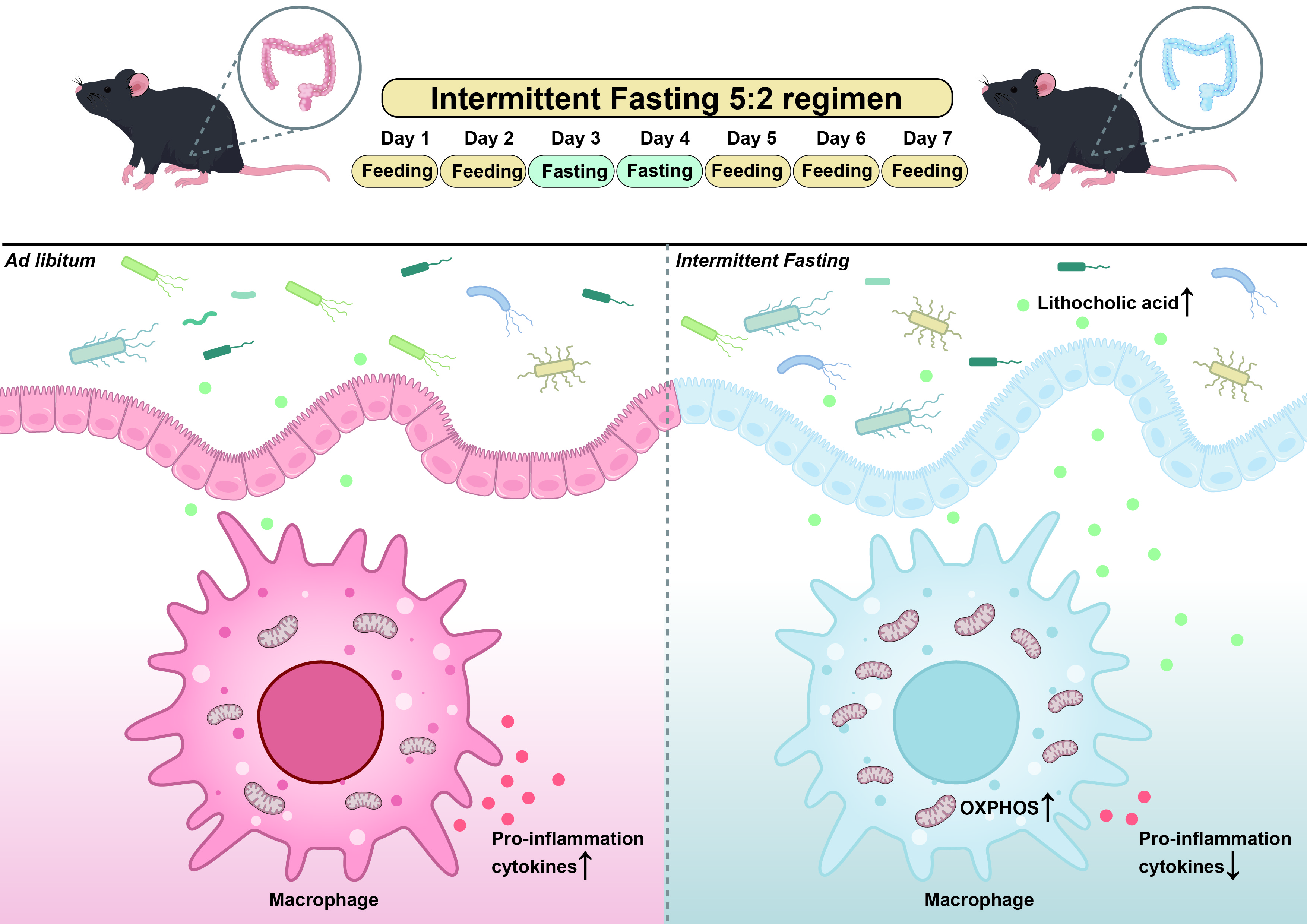

Supplement: Supplementary file 2 [file Image_1.JPEG]
